# Proceedings Buffalo Medical Association

**Published:** 1878-10

**Authors:** Joseph Fowler

**Affiliations:** Secretary


*ART. II.—Abstract of the Proceedings of the Buffalo Medical Association.* May 31, 1878. Reported by JOSEPH FOWLER, M. D., Secretary.

Members in attendance: Doctors J. F. Miner, Lothrop, White, Wyckoff, Rochester, Hauenstein, Bartlett, Wetmore, Brecht, Howe. S. S. Greene and Fowler.

The President, Dr. Lothrop presiding.

The regular order of business was suspended, to give Dr. J. F. Miner an opportunity to present a young man aged 19 years, who had recently dislocated his wrist. A month ago yesterday he fell down stairs, producing an injury of such a nature that the distal extremities of the bones of the forearm rested on the posterior portion of the carpus, which required a considerable force to reduce. This, the doctor said was one of the rarest dislocations in surgery, and demonstrates clearly that this dislocation does occur without fracture, though it has been denied by some surgeons.

Dr. Rochester said he knew the history of this patient, who had previously acted as his office boy.

He was seen soon after the accident by another physician, who told him his wrist was fractured, and proceeded to dress it by applying some kind of an apparatus, remarking that it would be necessary to break it over, to get it right.

He could detect no fracture, and referred him to Dr. Miner for treatment.

Dr. Fowler read a paper on rachitic disease—published elsewhere in Journal—When the reader had concluded, Dr. White arose, he said, to commend the paper, which had gone over the subject so fully, rather than attempt to add much thereto. It was one to which he had given much study in early professional life, and he found then as now, that there was much doubt as to its causation and pathology. There is nothing that strikes the American tourist in London so forcibly as the proportion of crooked legs he observes there; this deformity being vastly more common with them than with us. Why this should be so was difficult to explain, when we were surrounded by the same apparent influences which seem to be so fruitful in the production of the disease abroad.

Why Dr. Perry should have given as the result of his experience in Philadelphia, such a large percentage of rachitic patients, which nearly equalled that of London, he could not tell. Other practitioners in the same city fail to confirm his observations. Probably Dr. Perry was very enthusiastic upon the subject.

Dr. W. believes that locality has very much to do with its production. Why stone in the bladder is so frequent in some localities and almost unknown in others is a curious question to propound. The chemical theory, he thinks, a very unsatisfactory explanation of the disease. A treatment based upon this hypothesis generally failed to produce good results. The doctor believes the causes are external, and the treatment mentioned in the paper to be the proper one, viz: Hygienic care, tonics, good milk in abundance, etc.

Dr. Rochester said the disease was diffused in all classes. In England the usual diet differed very much from our own. The people in general eat very little meat. Why goitre is so common in certain localities could not be satisfactorily explained. Suspect that locality has something to do as a cause of rachitis. Dr. Perry was probably too enthusiastic, and that the symptoms he considered diagnostic of the presence of the affection, were not such as would warrant us in pronouncing the disease rachitis. Anything that produces cachexia is another element to be considered. We can often trace many cases to syphilitic and tuberculous parents. Think there may be something in race also, as there is a proneness of the Negro, to this form of disease. Deformity of the pelvis being much more frequent in the Negroes than in other races, but after all we believe the great cause to be found in locality.

Dr. Bartlett was of the opinion that the unfortunate condition of the London poor, their surroundings and habits differing so much from those of similar classes in our own country, might explain in a measure the great frequency of the disease among them. The dense fogs that envelope London, the limited amount of light the poor are allowed in consequence of the window tax imposed on them, the badly drained localities in which the poor reside, and the unwholesome food upon which they subsist, all probably combine to favor the production of the disease.

Dr. S. S. Greene said he desired to report a case of cerebro spinal meningitis. The patient was a young lady 18 years old. She was suddenly seized with convulsions, opisthotonus being present. She would throw herself about and bite her hands if not restrained. Gave sulph. Ether by inhalation, and chloral and morphine in full doses. Consciousness returned in about 48 hours when he gave 10 to 15 grains of quinine every four hours. Under its use she got quite well, and went to visit her grandmother when she had a relapse, her condition being as alarming as before. A similar treatment was pursued, and she has now almost entirely recovered. Dr. Greene said he would like to hear Dr. Rochester express his opinion of the case.

Dr. Rochester said he had known of the young lady's illness, having made visits in the same neighborhood at that time, and judging from the symptoms as given by Dr. Greene, he believed the case was one of hysteria, and not cerebro spinal meningitis; at least he believed many of the symptoms were hysterical.

Dr. Greene asked if it was common for hysterical patients to complain of pain in one limb.

Dr. Rochester replied, that hysteria simulated every known disease on the face of the earth.

Dr. Howe, chairman of the Executive Committee, reported that the following gentlemen had agreed to present papers at future meetings of the association:

July 2. Dr. Bartlett; subject, Accidental Hemorrhage.

August 6. Dr. Rochester; subject, Pulmonary Diseases of Elevator Employés.

September 3. Dr. Strong; subject, Practical Consideration concerning Puerperal Fever.

October 1. Dr. Bartow; subject, Influence of Alchohol upon the Liver.

November 5. Dr. Hartwig; subject, Modern Surgical improvements.

December 3. Dr. J. F. Miner; subject, The Diagnosis of Disease without Physical Signs.

On motion the report was adopted, and authority granted to have the same printed for distribution among the members.

Dr. Wetmore reported having seen during the week three cases of strangulated hernia, two of which he reduced by taxis, and the other he operated upon, liberating two knuckles of intestine and some omentum.

On motion, the Association adjourned.

